# Effect of Nocturnally Worn Occlusal Splints on Condylar Position

**DOI:** 10.1111/joor.70266

**Published:** 2026-07-12

**Authors:** Karl‐Heinz Utz, Jochen Feyen, Manfred Grüner, Helmut Stark

**Affiliations:** ^1^ Retired Königswinter‐Stieldorf Germany; ^2^ Department of Prosthodontics, Preclinical Education, and Dental Materials Science University Clinic Bonn Bonn Germany; ^3^ Retired Lennestadt Germany

**Keywords:** centric condylar position, condylar position, healthy participants, maximal intercuspal position, occlusal splints

## Abstract

**Background:**

Patients with craniomandibular dysfunction may experience changes in mandibular position over time when wearing occlusal splints continuously throughout the day and night. The aim of this study was to investigate whether similar changes can also be observed in healthy individuals who wear such splints exclusively at night.

**Methods:**

The study included 13 healthy participants who were provided with maxillary stabilization splints worn only during night‐time. Clinical evaluations were conducted after exactly 7 days, and the total study duration was 3 weeks. The maximal intercuspal position (MIP) as well as the centric condylar position (CR) were recorded in the condylar region of a measuring articulator. Additionally, the centric condylar position with the splint in place was determined several times using a thermoplastic registration material. The magnitude and direction of the positional differences were compared.

**Results:**

The differences in mandibular position between centric condylar position (CR, wax registration records) and maximal intercuspal position (MIP, no interdental records) measured 0.74 ± 0.35 mm at the first and 0.74 ± 0.37 mm at the final session. The corresponding distances between all CR records obtained with the splints and MIP were 0.82 ± 0.39 mm.

**Conclusion:**

Within the investigated sample, night‐time use of stabilization splints did not result in any change in mandibular position. A secondary finding was that occlusal adjustments were required during splint delivery, despite the use of facebows and registration procedures.

## Graphical abstract

Measuring articulator with electronic dial gauges, three‐dimensionally freely movable upper part and a 20 N weight. An occlusal splint was placed in the upper jaw with thermoplastic material as a centric relation record.

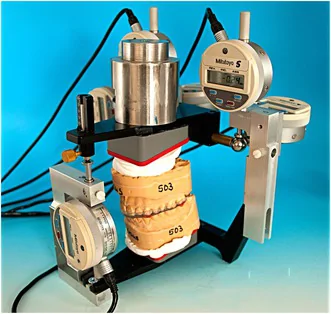

## 1 Background

Our clinical observations indicate that, following the insertion of occlusal splints worn continuously for 24 h per day, some—though not all—patients with complaints related to the masticatory system may exhibit changes in mandibular position over several weeks of treatment. A splint that has been carefully adjusted to the centric condylar position (CR), with uniform occlusal contacts and no slide upon firm jaw closure, may present with altered and uneven occlusal contacts after only a few days, despite identical occlusal control procedures. This suggests that the mandibular position, consequently the position of the condyles, has changed.

To anticipate such positional changes, pre‐treatment with occlusal splints has occasionally been recommended prior to initiating extensive prosthetic reconstructions. Some authors have assumed that, without this preliminary phase—that is, when jaw relation is determined and fixed dental prostheses are incorporated immediately—the mandibular position might change after final cementation. As a result, the newly fabricated restoration could lose its accurate interocclusal fit.

We therefore sought to determine whether comparable phenomena can also be observed in healthy, asymptomatic individuals who wear occlusal splints exclusively at night. This therapeutic approach typically represents the initial step in standard pain management for craniomandibular dysfunction, as for instance described by Peroz and Imhoff [1, 2]. Furthermore, it plays an important role in the recommendation to protect extensive ceramic restorations after placement by wearing a night guard during sleep [1, 2].

The null hypothesis of this study was that, in symptom‐free subjects, the position of the condyles in centric condylar position (CR) would remain unchanged during treatment with night‐time occlusal splints.

## 2 Methods

### 2.1 Random Sample

The participants were recruited from the staff of the university dental clinic in Bonn. Inclusion criteria required subjects to be stomatognathically healthy, defined as the absence of temporomandibular joint clicking, pain, or pronounced tooth wear. In addition, participants were required to present with a nearly complete dentition (wisdom teeth could be missing), a healthy periodontium and only a limited number of restorations (no more than four fillings or crowns per jaw).

Individuals who had worn occlusal splints within the previous 6 months were excluded from participation. The study protocol specified that participation would be discontinued if (1) unforeseen pain in the stomatognathic system occurred during the observation period or (2) existing restorations required replacement.

### 2.2 Protocol

#### 2.2.1 1st Session (Scheduled for 60 Min)

Participants were informed about the study procedures, and informed consent was obtained prior to inclusion (Ethics committee No. 0811). After recording the medical history, a clinical examination was performed.

Maxillary and mandibular impressions were taken using Rim‐Lock trays, adhesive varnish and machine‐mixed fast‐set alginate (Adhesive varnish, Alginoplast, Kulzer GmbH, Leipziger Straße 2, 63450 Hanau, Germany). The impressions were immediately transferred to the in‐house dental laboratory for further processing.

For the subsequent examinations, the treatment chair was adjusted to the zero position (semi‐recumbent), with Camper's plane oriented such that the head was reclined approximately 30 degrees dorsally relative to the floor. Arbitrary Reference facebows (Gamma Dental GmbH, Wasserzeile 35, 3400 Klosterneuburg, Austria) and silicone modelling material applied to the bite forks (Finopaste, Fino GmbH, Mangelsfeld 18, 97708 Bad Bocklet, Germany) were used to prepare the casts for skull‐related transfer into the articulators.

For each participant, three individually trimmed, double‐layer wax wafers (Beauty Pink X‐hard wax; Riss Dental GmbH, Hochstädter Landstraße 109, 63454 Hanau, Germany) were used to record the centric condylar position. The wafers were warmed in a water bath at 50°C and, in cases of pronounced overbite, trimmed mesially to the canines if necessary.

##### 2.2.1.1 Procedure in the Dental Laboratory After the First Session (Scheduled for 260 Min)

- The impressions were poured in type IV dental stone (Fuji Rock; GC Germany GmbH, Seifgrundstraße 2, 61 348 Bad Homburg, Germany) and allowed to set for 30 min. The first pair of casts was then prepared with a magnetic split‐cast system in the maxillary model. A second pair of casts was duplicated no earlier than 12 h later using addition‐curing silicone in metal cuvettes against the secondary bases of the original casts.
- Dental technicians mounted the original maxillary casts in previously synchronized (calibrated) Reference articulators using the facebow transfer. The opposing mandibular casts were subsequently mounted in centric relation (CR) by the two dentists, each using one of the three wax records for their respective participants (Figure 1).
- The dental technicians then mounted the duplicate casts alternately using the same CR record that had initially been used by the dentists to mount the mandibular casts in the articulators. The objective was to position the duplicate casts in exactly the same geometric relationship as the original casts within the articulators.
- Prior to fabrication of the occlusal splints, the duplicate maxillary casts were surveyed to determine undercuts in the intended path of insertion (prosthetic equator). The vertical dimension at the incisal pin was adjusted to a maximum of 1 mm above the first interocclusal contact of the casts in CR, corresponding approximately to the vertical increase produced by the wax records.
- The maxillary occlusal splints were fabricated on a 1 mm Miniplast baseplate and subsequently adjusted occlusally in the articulator using autopolymerizing resin (Palapress; Kulzer GmbH, Leipziger Straße 2, 63450 Hanau, Germany). The design followed the fundamental principles of a Michigan splint [3], but without freedom in centric. Accordingly, the splint provided a clearly defined centric relation, a flat occlusal surface in the posterior region, one to two contact points for each mandibular posterior tooth via the vestibular cusp tips and a flat anterior guidance with front canine protection, allowing limited posterior disclusion during eccentric movements.
- Occlusal adjustments were performed in the articulator using wide articulating foil (Hanel foil; Coltène/Whaledent GmbH, Raiffeisenstraße 30, 89129 Langenau, Germany) without removing the splints from the casts at this stage. The splints were then trimmed approximately 1 mm apical to the marked prosthetic equator, removed from the casts and finished. The intended result was a slightly retentive yet passive fit.

**FIGURE 1:**
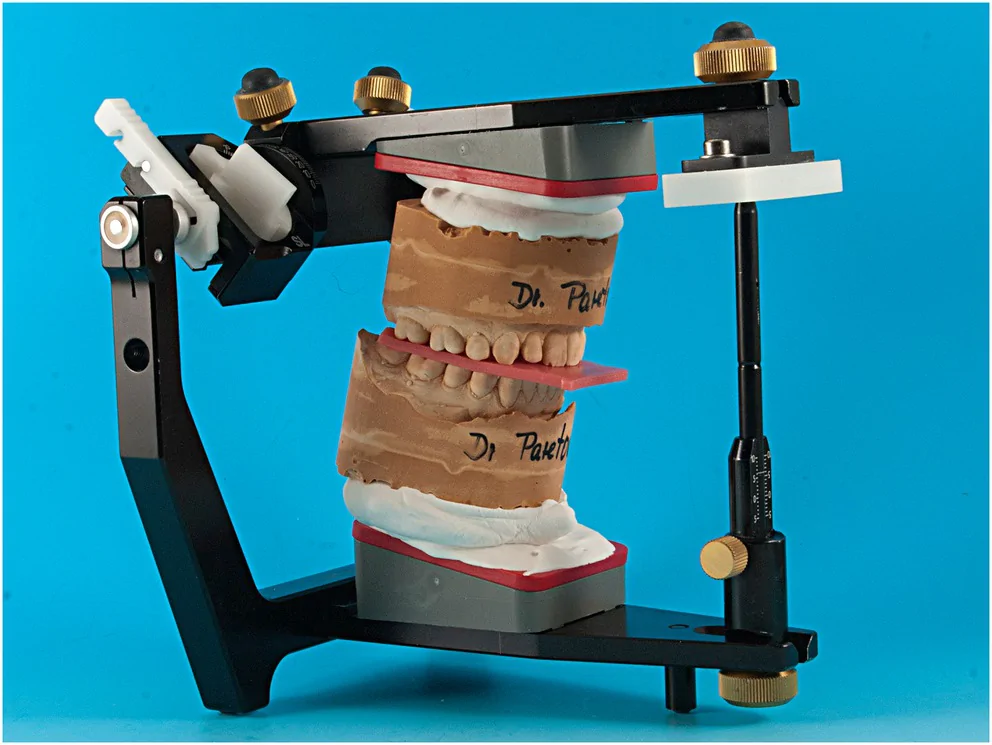
After mounting the upper jaw with a medium‐value Reference facebow, the lower jaw was aligned with a centric wax record in a calibrated Reference articulator.

##### 2.2.1.2 Measuring Articulator

- The measuring articulator consisted of a modified Reference articulator with a three‐dimensionally freely movable upper part. Spherical contact elements (metal balls) were mounted bilaterally in the condylar regions. Three electronic digital dial gauges on the right side and two on the left side were attached to the lower part of the articulator and oriented along the three spatial axes (Digimatic dial gauge ID‐C, Model 543; Mitutoyo Deutschland GmbH, Borsigstraße 8–10, 41 469 Neuss, Germany). These gauges were in contact with the metal balls representing the condyles. An additional dial gauge was installed in the incisal pin region; however, this measurement was not included in the present evaluation. During individual measurements, the upper part of the articulator was loaded with a constant force of 20 N (Figure 2).
- On each test day, the dial gauges were calibrated to zero in the morning using an adjustment block positioned between the upper and lower members of the measuring device. This procedure ensured that potential positional changes of the gauges—such as those caused by battery replacement or mechanical impacts—were compensated for and allowed direct comparability of the measurements obtained on different test days.

**FIGURE 2:**
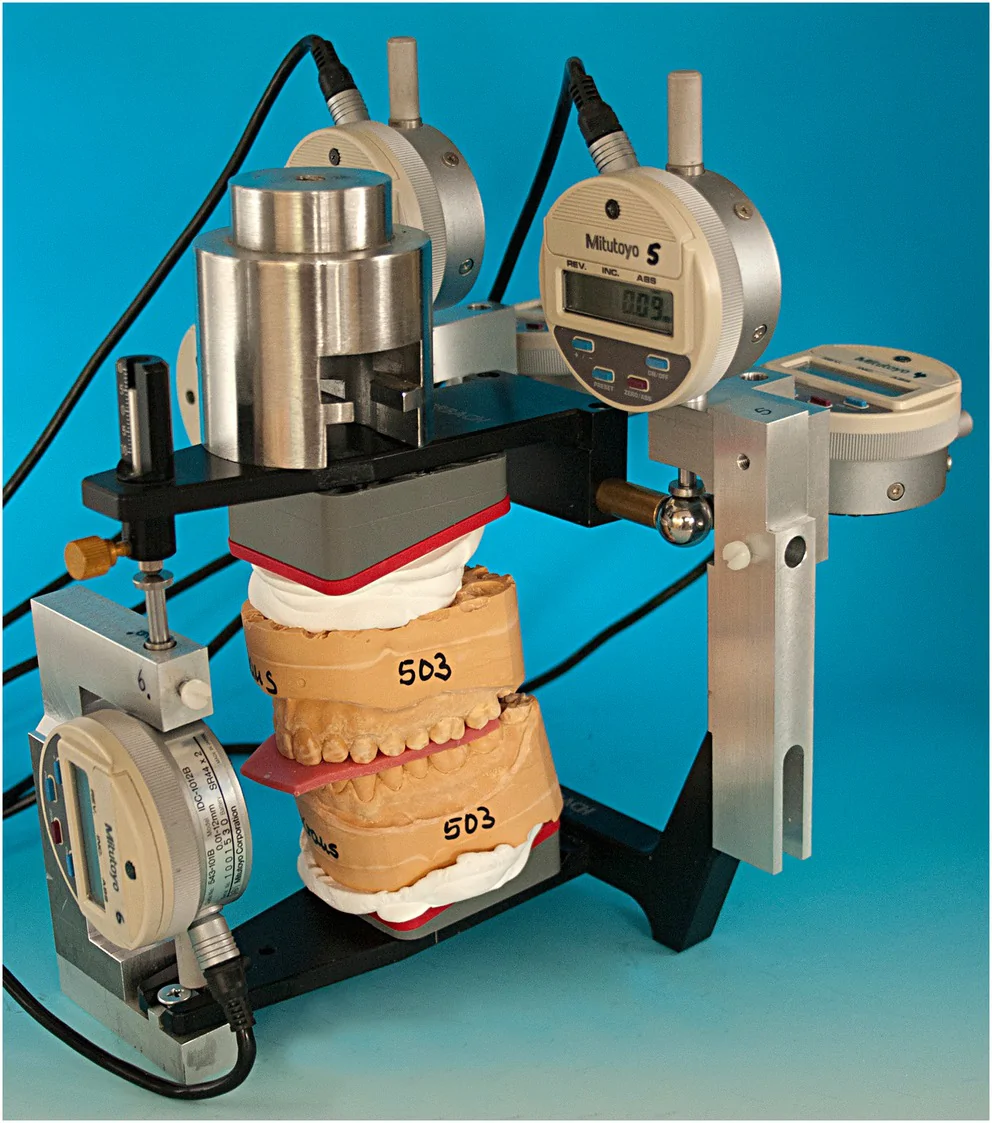
Self‐constructed measuring articulator with electronic dial gauges, three‐dimensionally freely movable upper part and a 20 N weight. A centric wax record was placed between the original casts for measurement.

##### 2.2.1.3 Measurements After the First Session

- All measurements were performed by the two clinicians. First, the original casts mounted in the articulator were transferred to the measuring articulator. The three wax wafer records were each measured twice after reassembling the cast‐registration complex (a, b). These measurements were labelled 1a, b to 3a, b. They were used to determine the centric relation (CR) recorded in the test persons at the beginning of the study (CR reproducibility 1, initial measurement; Table 1).
- Subsequently, the original casts were brought into a ‘manually adjusted’ intercuspal position without the use of a registration record. The casts were guided against each other and held in the most stable intercuspal position, following the procedure commonly used when mounting casts in maximal intercuspal position (MIP). These positions were then measured (measurements 4a, b) in order to determine the reproducibility of the maximum intercuspal position (MIP reproducibility 1, initial measurement; Table 1).

**TABLE 1:** The individual examination sessions and the nature and significance of the measurements.

Session No.	No. Measurements	What was measured?	Significance of the measurements
1 (Start)	1, 2, 3	CR 1, wax records	Reproducibility CR wax record, initial measurement
	4	MIP 1, ‘manually jogged’ intercuspal occlusion	Reproducibility MIP, initial measurement
2 (Start)	5	CR thermoplastic material on splint 1	Reproducibility CR splint, initial measurement
	6	CR thermoplastic material on splint 2	Reproducibility CR splint, control measurement
3 (Control)	7	CR thermoplastic material on splint 3	Reproducibility CR splint, control measurement
4 (Control)	8	CR thermoplastic material on splint 4	Reproducibility CR splint, final measurement
5 (End)	9, 10, 11	CR 2, wax records	Reproducibility CR records, final measurement
	12	MIP 2, ‘manually jogged’ intercuspal occlusion	Reproducibility MIP, final measurement

#### 2.2.2 2nd Session (Scheduled for 60 Min)

- The first occlusal contact between the antagonistic pairs of teeth during closure of the mandible in centric condylar position was identified intraorally using articulating foil. The findings were documented in an occlusal protocol, after which the occlusal splint was inserted, with particular attention paid to achieving a rock‐free fit.
- The occlusion on the splint was then carefully adjusted to the centric condylar position. Antagonistic contacts on the splint were designed such that, when the mandible was gently guided into the centric condylar position (Dawson's‐grip [4]), simultaneous and evenly distributed interocclusal contacts occurred on both the right and left sides at the moment of first contact with the splint. Subsequent firm biting by the participants should not result in any further change in mandibular position. The front and canine protected articulation was adjusted to provide a flat and smooth guidance (Figure 3).
- A centric relation record was then obtained under the same guidance conditions using a thermoplastic registration material applied to the splint in the regions of the second molars and first premolars (Bite Compound; GC Germany GmbH; see above) (Figure 4).

**FIGURE 3:**
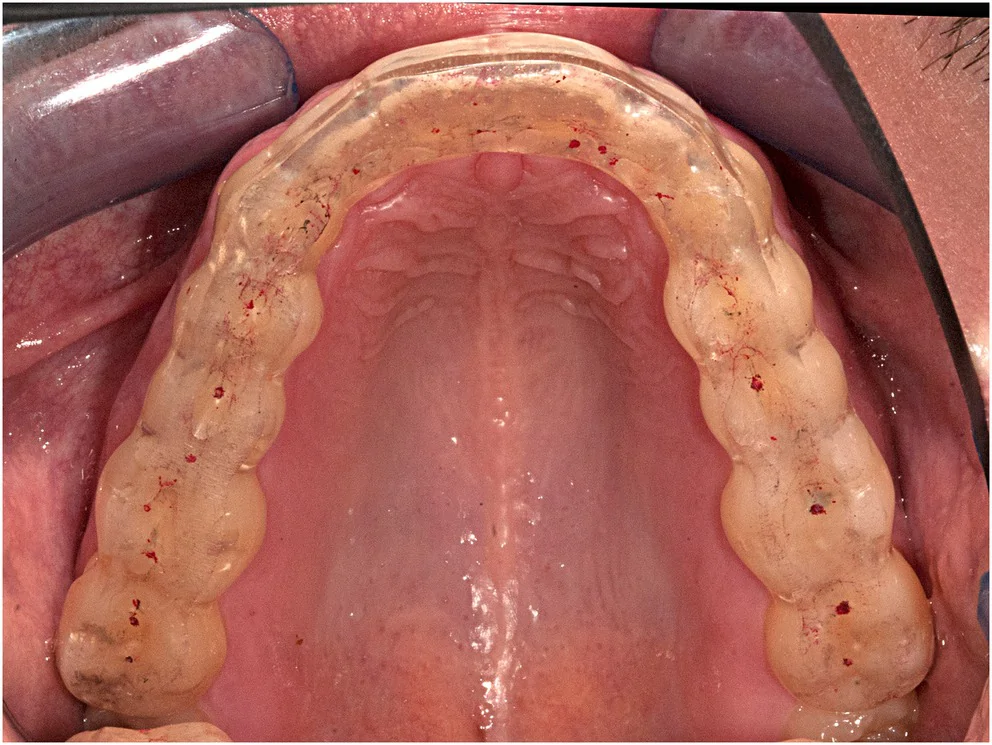
Upper jaw occlusal splint with flat occlusal relief and front‐canine protected articulation used in the sample. We ensured that each tooth had at least one interocclusal contact.

**FIGURE 4:**
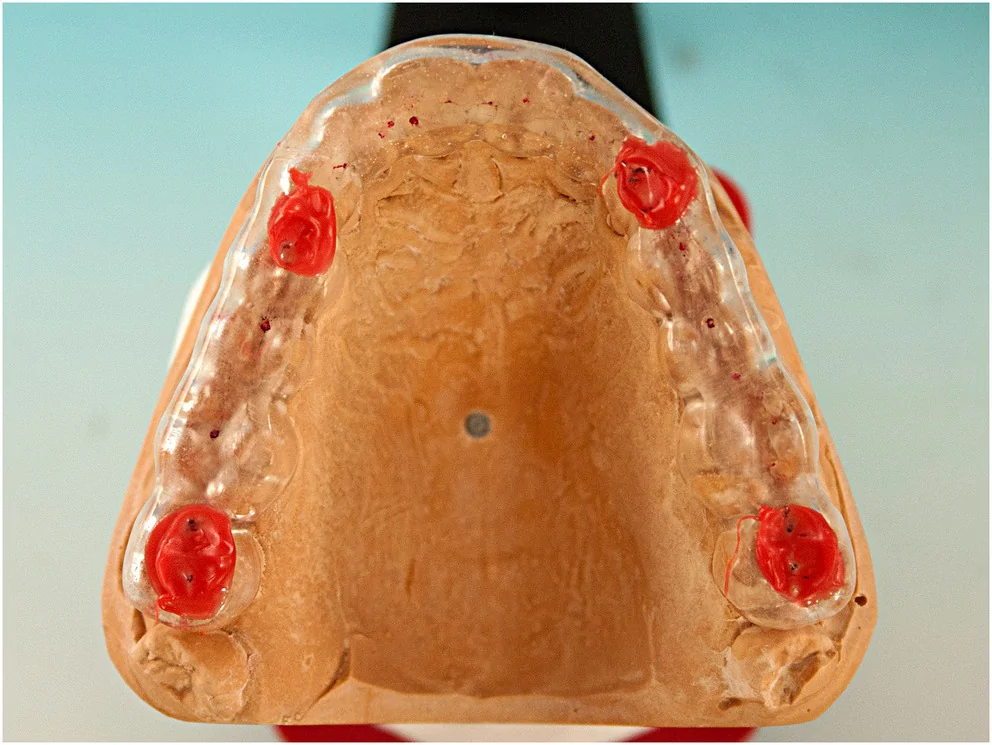
Occlusal splint in the upper jaw with thermoplastic material as a centric relation record.

##### 2.2.2.1 Measurements After the Second Session

- The splint with the integrated thermoplastic record was placed on the original casts in the measuring articulator, and the resulting splint position was measured. As in the previous procedures, the measurement was performed twice consecutively (measurements 5a and 5b). The purpose of these measurements was to document the centric relation that had been clinically adjusted on the splint at the beginning of the study (CR splint 1, baseline measurement; Table 1).
- The thermoplastic stops were then carefully removed from the resin, and the splints were inserted intraorally.
- The participants received detailed instructions regarding splint use. They were asked to record their subjective sensations each evening and morning. The splint was to be worn every night and, on the seventh and final day, continuously until the follow‐up examination, including during daytime.

#### 2.2.3 3rd and 4th Sessions (Each Scheduled for 45 Min)

- The third clinical examination—like all subsequent examinations in the study—was conducted exactly 7 days after the second session.
- During this follow‐up visit, participants were asked about their experiences with the occlusal splint.
- Apart from this inquiry, all examination procedures were identical to those performed during the second session.

##### 2.2.3.1 Measurements After the 3rd and 4th Sessions

- The measurements obtained during the third session were labelled 6a and 6b, and those from the fourth session were labelled 7a and 7b. These corresponded to the measurements performed during the second session, representing the position of the condylar spheres with the centric relation splints (CR splints 2 and 3; control measurements; Table 1).

#### 2.2.4 5th Session (Scheduled for 45 Min)

- The clinical procedures were initially identical to those performed in sessions 3 and 4.

##### 2.2.4.1 Measurements After the 5th Session

- Centric relation (CR) was again recorded using thermoplastic compound applied to the splints (measurements 8a and 8b; Table 1). Measurements 5 to 8 represent the reproducibility of CR recorded on the splints.
- Using three additional CR wax records (measurements 9a, b to 11a, b), the centric condylar positions were determined once more (CR reproducibility 2, final measurement; Table 1).
- Finally, the original casts were reassembled in a manually guided intercuspal occlusion. The maximal intercuspal position (MIP) at the end of the study was determined using measurements 12a and 12b (MIP reproducibility 2, final measurement; Table 1).

### 2.3 Statistical Analysis

Measurements a and b were summed and their mean value was calculated. This mean represented the reproducibility of the positioning of the casts relative to each other in the maximal intercuspal position (MIP), in the wax records, or on the splints. All subsequent calculations were based on the corresponding mean values derived from measurements a and b.

For the spatial calculation of the condylar positions obtained in measurements 1–3, 5–8 and 9–11, the resultant value was calculated from the three spatial directions: anterior–posterior (x), cranial‐caudal (vertical; y) and transverse (z).

The computer‐recorded measurements were analysed using custom‐developed software. Owing to the limited number of participants, the data were evaluated only descriptively using IBM SPSS 21 Statistics, provided by the computing centre of the University of Bonn.

## 3 Results

### 3.1 Random Sample and Incidental Findings

Thirteen participants (10 female, 3 male) completed all study sessions. The mean age was 26.6 years (range: 24–35 years).

When the mandible was gently guided into the centric condylar position without a splint, the initial interocclusal contact was most frequently observed between the premolars on the left side. In only three cases was the first contact located on the right side. Only one participant showed no clinically detectable difference between the centric condylar position and the maximal intercuspal position (MIP).

After insertion of the splints, the first contacts in the centric condylar position occurred twice in the anterior region, five times on the right side and six times on the left side. Occlusal adjustment of these premature contacts to achieve evenly distributed contacts required the greatest amount of time during the initial integration of the splints. At each of the three subsequent follow‐up visits, the time required for these adjustments progressively decreased:

**Table:** 

2nd session	12 min (range: 7–23 min)
3rd session	5 min (range: 0–12 min)
4th session	3 min (range: 1–10 min)
5th session	3 min (range: 0–8 min).

### 3.2 Sensations Experienced by Subjects When Wearing the Occlusion Splints

Participants wore the splints for a mean duration of 7.8 h per night (range: 7.0–8.5 h). None of the participants reported sleep disturbances or pain during the period of splint use.

Subjective feedback regarding splint wear was predominantly described as ‘unobtrusive’ or ‘no particular observations’. All participants reported that, after self‐inserting the splints in the evening, they were able to find the intended splint position without difficulty.

One participant reported during a follow‐up visit that, after removing the splint in the morning, the maxillary and mandibular teeth initially did not fit together well and that the interocclusal contacts appeared altered. However, this sensation was only once and transient.

Additional comments reported by individual participants included: ‘the splint feels tight’ (reported twice), ‘the splint presses on the teeth’, ‘the splint interferes with speech’, ‘annoying’, ‘the lower jaw tends to move forward’, ‘uncomfortable sensation in the posterior teeth on the first day’, ‘foreign body sensation’, ‘dry mouth’ and ‘the splint makes me feel more relaxed when I am stressed’.

### 3.3 Results of the Measurements

J. F. examined six; K.‐H. U. seven subjects.

The *reproducibility of the centric condylar position (CR)* in the study sample was determined in the condylar region of the measuring articulator and expressed as three‐dimensional spatial deviations (in millimetres):

- With double wax wafer records 1st session, 3 records a, b (Nos. 1–3)

**Table:** 

J. F.	R 0.41 ± 0.14	L 0.45 ± 0.14	(0.22–0.64)
K.‐H. U.	R 0.31 ± 0.15	L 0.51 ± 0.16	(0.09–0.93)
Total	R 0.37 ± 0.14	L 0.52 ± 0.26	(0.09–0.93)

- With double wax wafer records 5th session, 3 records a, b (Nos. 9–11)

**Table:** 

J. F.	R 0.51 ± 0.19	L 0.45 ± 0.17	(0.22–0.78)
K.‐H. U.	R 0.34 ± 0.15	L 0.58 ± 0.33	(0.17–0.68)
Total	R 0.41 ± 0.20	L 0.48 ± 0.16	(0.17–0.78)

- Combined result with double wax wafer records from the right and left sides of both examiners (rounded): 0.45 ± 0.19 mm (range: 0.09–0.93 mm)
- With records on the upper jaw splints, 4 records a, b (Nos. 5–8)

**Table:** 

J. F.	R 0.70 ± 0.18	L 0.67 ± 0.33	(0.27–1.13)
K.‐H. U.	R 0.43 ± 0.19	L 0.44 ± 0.20	(0.17–0.82)
Total	R 0.55 ± 0.23	L 0.52 ± 0.29	(0.17–1.13)

The *reproducibility of the maximal intercuspal position (MIP)* (‘manually guided intercuspal occlusion’) between the original casts in the fifth session, as determined by the clinicians (measurement No. 12), was expressed as spatial deviations in millimetres:

**Table:** 

J. F.	R 0.08 ± 0.06	L 0.09 ± 0.06	(0.01–0.15)
K.‐H. U.	R 0.08 ± 0.05	L 0.11 ± 0.07	(0.01–0.20)
Total	R 0.08 ± 0.05	L 0.10 ± 0.06	(0.01–0.20)

The results for the two clinicians are presented together below, as the individual reproducibility values reported above were comparable in magnitude.

The calculated *differences between the mandibular positions in centric condylar position (CR) and in the maximal intercuspal position (MIP)* at baseline, during the follow‐up examinations, and at the end of the observation period allow assessment of whether a clinically measurable shift in mandibular position occurred after splint therapy:

- Measurements CR 1 wax (Nos. 1–3, start session) to MIP 1 + 2 (Nos. 4 + 12) spatially (Figure 5):

**FIGURE 5:**
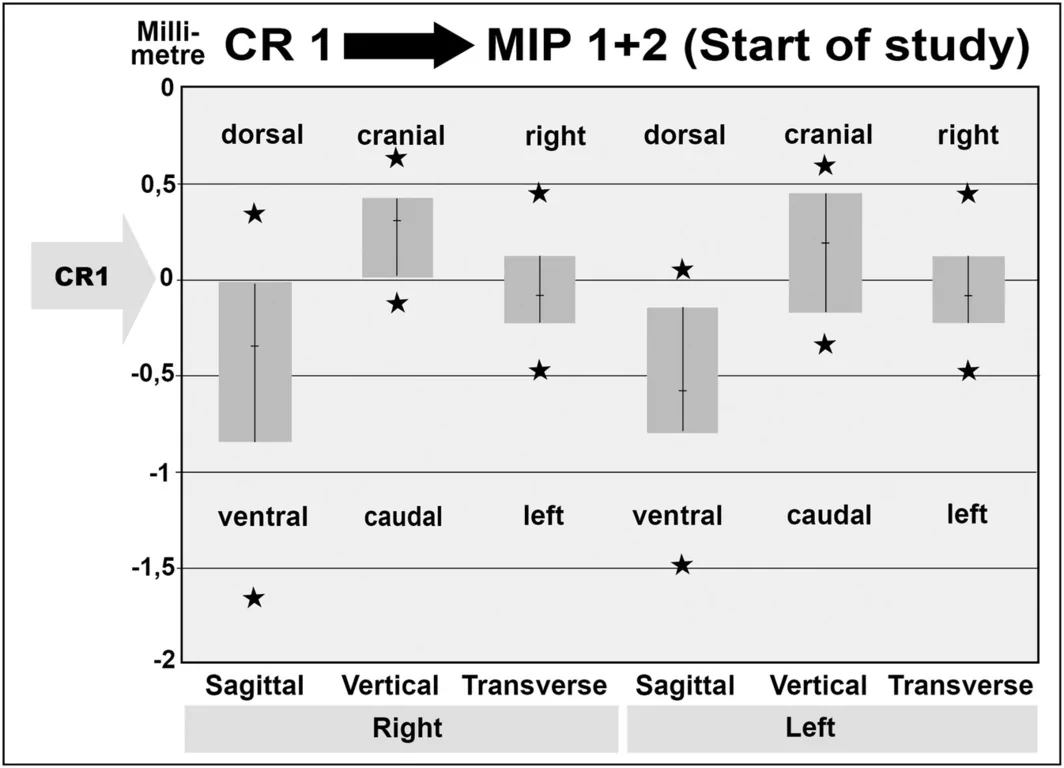
The deviations in the individual spatial directions between the CR 1 and the MIP (whisker plot: Median values, interquartile range, extremes) determined with the aid of the wax records in the first session (= start of the study). The left transverse values were obtained from the right dial gauge. On average, the MIP is slightly caudal and anterior to the CR. CR = centric relation; MIP = maximal intercuspal position.

**Table:** 

Right	Median 0.68	Mean ± SD	0.71 ± 0.36	Range	(0.30–1.63)
Left	Median 0.69	Mean ± SD	0.76 ± 0.33	Range	(0.22–1.45)

- Measurements CR 2 wax (Nos. 9–11, final session) to MIP 1 + 2 (Nos. 4 + 12) spatial (Figure 6):

**FIGURE 6:**
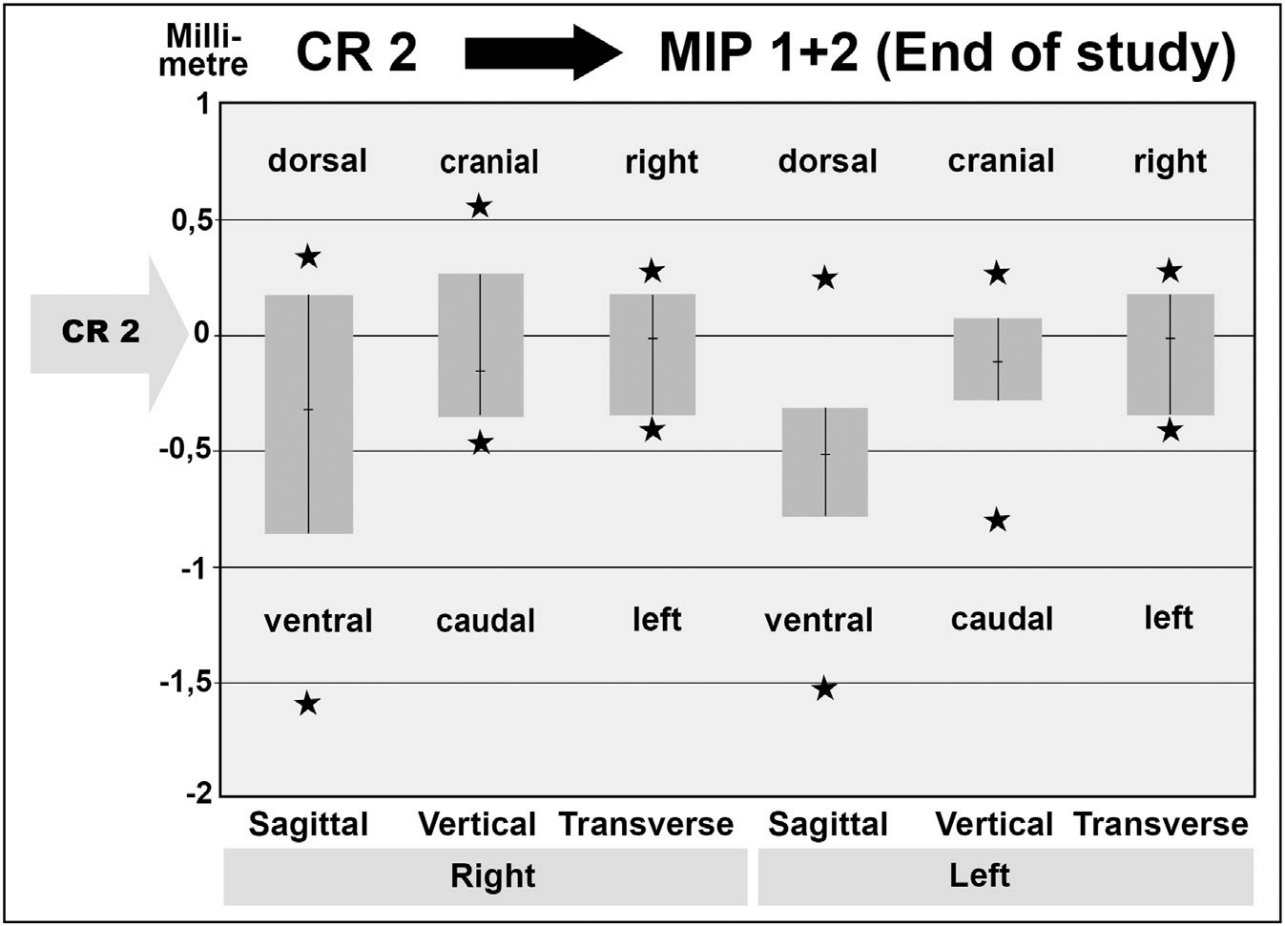
The deviations in the individual spatial directions between the CR 2 and the MIP (whisker plot: Median values, interquartile range, extremes) determined using the wax records in the 5th session (= end of the study 3 weeks later). The left transverse values were obtained from the right dial gauge. On average, the MIP is slightly caudal and anterior to the CR.

**Table:** 

Right	Median 0.65	Mean ± SD	0.74 ± 0.38	Range	(0.30–1.63)
Left	Median 0.61	Mean ± SD	0.74 ± 0.35	Range	(0.33–1.58)

- Measurements MIP 1 + 2 (Nos. 4 + 12) for CR splints (Nos. 5–8 controls and final session), spatial (Figure 7):

**FIGURE 7:**
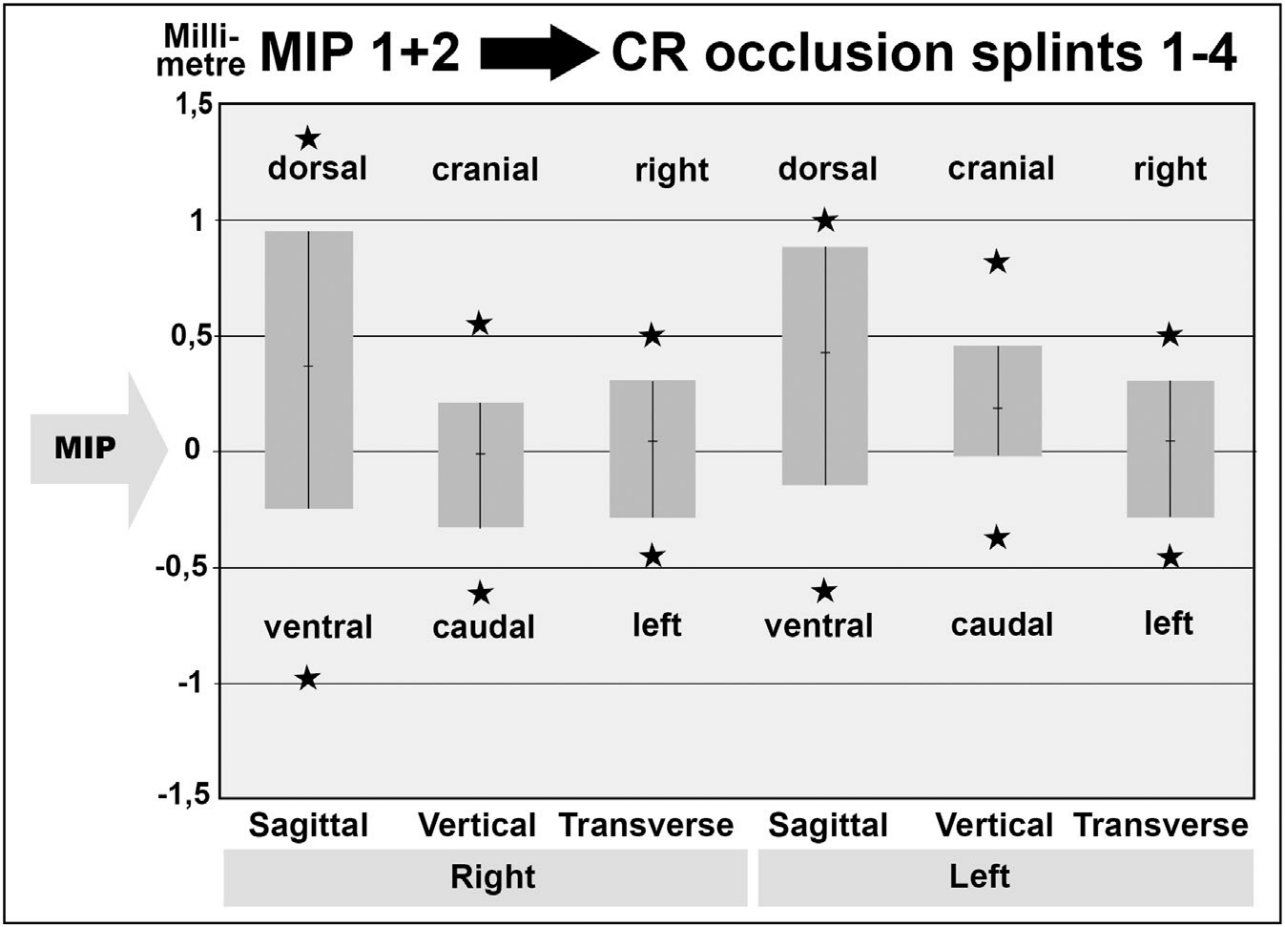
All CR positions on the splints were compared with the MIP positions in the individual spatial directions in this representation (whisker plot: Median values, interquartile range, extremes). In contrast to Figures 5 and 6, the starting point here is the MIP. The left transverse values were obtained from the right dial gauge. On average, the CR is slightly cranial and dorsal to the MIP.

**Table:** 

Right	Median 0.86	Mean ± SD	0.84 ± 0.35	Range	(0.37–1.38)
Left	Median 0.69	Mean ± SD	0.79 ± 0.43	Range	(0.36–1.78)

The measurement results of the control sessions (third and fourth sessions) are not presented separately, as they were within the same range and were already included in the above calculation (measurements 5–8).

Figures 5, 6, 7 illustrate the deviations in the individual spatial directions.

## 4 Discussion

The spatial distances between the mandibular positions in the centric condylar position (CR) and the maximal intercuspal position (MIP) demonstrate very good agreement, measuring 0.74 ± 0.35 mm in the first session (Figure 5) and 0.74 ± 0.37 mm in the fifth session (Figure 6).

The deviations between the maximal intercuspal position and the centric condylar positions recorded on the occlusal splints, which served as control values, are within a comparable range (0.83 ± 0.39 mm), as are the values obtained in all intermediate control measurements. Deviations in the individual spatial directions are likewise comparable. In addition, the participants generally reported no occlusal discomfort upon removal of the splints in the morning.

Consequently, the null hypothesis formulated for this study cannot be rejected: In healthy participants, treatment with splints worn exclusively at night does not result in a change in the position of the condyles in the centric condylar position. This finding is also significant for the fabrication of bite splints following the placement of extensive all‐ceramic restorations, as these are considered standard practice for protecting restorations against fractures and chipping [1, 2].

By comparing the distances and spatial directions between the centric condylar position (CR) and the maximal intercuspal position (MIP) at the beginning and at the end of the study, it can be determined whether the mandibular position changed as a result of wearing the splints at night. The repeatability of the individual registrations is critical for interpretation, as it defines the magnitude of change (i.e., distances in millimetres) at which a meaningful interpretation is possible.

With regard to the accuracy of the maximal intercuspal position, earlier investigations using the exactly same methodology and a significantly larger sample reported a value of 0.10 ± 0.05 mm (range: 0–0.51 mm) [5]. The values determined in the present study—0.09 ± 0.06 mm (range: 0.01–0.20 mm) are within the same range.

Using the same methodology, the accuracy of recording the centric condylar position (CR) with unrefined wax wafer records in subjects with healthy dentition was reported to be 0.33 ± 0.08 mm (range: 0.02–1.37 mm) in the joint region [6, 7]. In the present study, the precision of determining the centric condylar position was lower, at 0.45 ± 0.19 mm (range: 0.09–0.93 mm). This difference is likely attributable to the smaller sample size.

For interpretation of the present results, the accuracy of CR recordings obtained with thermoplastic material on the splints should also be considered. This value was 0.54 ± 0.26 mm (range: 0.17–1.13 mm) and thus lies within the range reported for CR determination using wax wafer records.

To assess whether the intended mandibular position in the centric condylar position (CR) had been achieved on the wax wafer ~ or splint records during the final phase of occlusal adjustment, the tactile sensitivity of the participants was also utilized [8]. This approach likely contributed to the high level of comparability of results between clinicians [7].

The distances between CR and MIP determined in the present sample (0.74 ± 0.37 mm) are consistent with the findings of a comprehensive evaluation conducted in a separate, significantly larger sample, which reported a value of 0.86 ± 0.52 mm [9].

To date, three‐dimensional assessment of mandibular condylar position before and after occlusal splint therapy has been performed predominantly in patients with craniomandibular disorders. The methods applied have mainly included cone‐beam computed tomography (CBCT) and other temporomandibular joint imaging techniques [10, 11, 12, 13, 14, 15, 16, 17, 18, 19], lateral teleradiographic imaging [20], magnetic resonance imaging [21, 22, 23] and direct three‐dimensional electronic measurements [22, 24, 25, 26, 27]. In addition, changes in the centric hinge axis [28] and positional changes recorded during central bearing tracing procedure [29] have been investigated. Healthy subjects were also included in several studies [22, 24, 25, 26, 27, 28], either as study participants or as control groups [12, 13, 15, 20, 23]. Ahmed et al. 2023 [10] and Hasegawa et al. 2011 [23] specifically investigated occlusal splints worn exclusively at night. The majority of authors—and the conclusions of a recent systematic review [30]—reported a tendency for the mandibular position to shift anteriorly and inferiorly following splint therapy [10, 11, 13, 17, 18, 19, 22, 24, 25, 29]. Imaging techniques are undoubtedly highly accurate; moreover, unlike indirect measurements in an articulator, they allow for direct assessment of condylar position.

None of the studies published to date are directly comparable with the indirect measurements performed in the articulator in the present investigation. The measurement techniques employed, as well as the requirements regarding splint wear time and the health status of the stomatognathic system, differ substantially across studies. Moreover, most studies do not report data on the accuracy of repeated measurements, either within the same situation or the same day or across different days. As demonstrated by the findings of the present sample, however, the reproducibility of the methods used is of central importance for the interpretation of the results.

The clinical determination of the centric condylar position is the critical factor in this study, as this is a biological limit position of the temporomandibular joints and not—as in the case of the maximal intercuspal position, which can be determined much more precisely—a position determined by the hard tooth substance. Based on the calculated reproducibility, we conclude that deviations of less than approximately 0.5 mm are not considered significant. Under this assumption, all parameters measured at the beginning and end of the study showed comparable values. The directions of the deviations between CR and MIP in the individual sessions also allow for this interpretation and do not appear to have changed either (Figures 5, 6, 7). We therefore conclude that the mandibular position of the healthy subjects did not change as a result of wearing the occlusal splints at night.

In a similar manner, it would now be necessary to examine whether this also applies to patients suffering from craniomandibular dysfunction.

A side observation relevant to clinical practice is that occlusal splints fabricated in a semi‐adjustable articulator—even after facebow transfer and careful registration of the centric condylar position—often do not fit precisely when inserted in the patient's mouth and therefore require occlusal adjustment. This was also observed in the present study, despite both clinicians having extensive experience with the registration procedures.

Consequently, it can be assumed that this also applies to the cementation of fixed dentures, which corresponds to the authors' practical experience. The extent to which such dentures can be fitted without occlusal corrections after complex and particularly careful registration, and whether the occlusion can initially be left unchanged for a short period in order to be corrected promptly during a responsible follow‐up examination, cannot be answered by this study and requires further investigation.

We would have preferred the occlusal splints to be worn throughout the entire day. However, it was not possible to recruit a sufficient number of participants for the planned, very time‐consuming examination protocol because no remuneration was provided. The ethics committee had restricted the selection of participants, as we were not permitted to include students in the study due to concerns about dependency relationships. The study design therefore had to be adapted. Another limitation is the small sample size and the unbalanced gender distribution, which ultimately led to the decision not to perform further statistical analyses. True randomization and blinding were not implemented.

Due to the effort involved, the study was limited to 3 weeks. However, we do not believe that a longer study period would have yielded changed results, as no metric differences were observed when comparing the weekly check‐ups. As we deliberately investigated healthy subjects, we cannot draw any conclusions as to whether different results would be obtained in patients with temporomandibular disorders (TMD).

We would also like to point out that, with regard to conclusions concerning ceramic restorations, no fully restored patients were examined; instead, healthy subjects were investigated.

If the occlusal plane in the articulator is strongly tilted, the placement of the records is significantly more prone to error and associated with lower measurement accuracy.

The splints were measured on the original casts, whereas their fabrication was carried out on duplicate casts. The reason for this procedure was that the original casts had to be protected from fractures and wear, as they were still required for measurements until the final session. This approach may have introduced inaccuracies; however, these are included in the results. Finally, it should be noted that the actual displacements of the mandibular condyles were not recorded. Instead, the movements of articulator condyles with a transverse distance of 110 mm were measured.

Arbitrary facebows were used in order to reduce the effort required from both the participants and the investigators. The error that occurs in comparison with individual terminal hinge axes was considered acceptable because the vertical difference between the splints and the wax registration records was certainly small. However, this lowering error is included in the results for the distances between CR and MIP.

The occlusal splints were to be worn until the examination appointment on the respective examination day, which resulted in varying wearing times on that day, for example in participants who had appointments in the afternoon.

## 5 Conclusions

If occlusal splints are fabricated after registration of the centric condylar position and produced with a facebow in a semi‐adjustable articulator, occlusal adjustment is still necessary when fitting them to the patient.

We further conclude that, in asymptomatic young individuals undergoing short‐term nocturnal splint therapy, no pre‐treatment with occlusal splints seems to be necessary prior to the insertion of fixed restorations, as no change in the position of the mandible is to be expected after insertion. These findings are also relevant for occlusal protective splints intended to prevent fractures and chipping following the placement of extensive ceramic restorations.

## Author Contributions

**Karl‐Heinz Utz:** conceptualization, investigation, writing – original draft, formal analysis, project administration. **Jochen Feyen:** conceptualization, project administration, formal analysis, methodology. **Manfred Grüner:** investigation, visualization, project administration. **Helmut Stark:** conceptualization, writing – original draft, validation.

## Ethics Statement

Ethics committee Bonn, ethics committee, Running No. 0811 (05.07.2010).

## Consent

The authors declare that the test subjects gave their written consent to the examination.

## Conflicts of Interest

The authors declare no conflicts of interest.

## Data Availability

The data that support the findings of this study are available from the corresponding author upon reasonable request.
